# User-Centered Design of Limb Prostheses: A New University Course Designed to Spark Interest in Orthotics & Prosthetics for Bioengineering Students

**DOI:** 10.33137/cpoj.v6i2.41789

**Published:** 2023-12-22

**Authors:** G Fiedler, J Samosky

**Affiliations:** 1 Department of Rehabilitation Science and Technology, School of Health and Rehabilitation Sciences, University of Pittsburgh, Pittsburgh, United States.; 2 Department of Bioengineering, Swanson School of Engineering, University of Pittsburgh, Pittsburgh, United States.

**Keywords:** User-Centered Design, Design Thinking, 3D Printing, Prototyping, Education, Outreach, Professional Development

## Abstract

There is a current need to increase recruitment in orthotics and prosthetics, and a promising approach is to increase awareness, interest and cross-disciplinary engagement in O&P among students of allied disciplines such as bioengineering. We describe a new interdisciplinary course we jointly developed at the University of Pittsburgh and deployed for the first time in the spring of 2023. The course was built on core foci of human-centered design, design thinking, experiential learning, 3D printing, creative problem-solving and prototyping. We leveraged a real-world project-based learning approach that included early and ongoing involvement of student teams with clients who used prosthetics. We explored creating a learning environment in which bioengineering students were motivated to learn about the O&P field by partnering with clients to investigate their unmet prosthetic needs and invent new solutions, with computer-aided design and 3D printing as key enabling technologies. Each student team produced an individually designed and fitted device for a specific application for a person with limb difference. Student feedback was positive throughout with several recipients expressing enthusiasm about the field of O&P and about the opportunity to work with actual patients. Several students stated their new-found interest in pursuing a career in the field. We believe that this sort of class offering could be implemented in many institutions that host O&P graduate programs, to raise awareness of the profession and attract more and better prepared applicants.

## INTRODUCTION

Much excitement has accompanied the rise of 3D printing and the associated benefits for low-cost rapid prototyping. There is no shortage of promising applications for this technology, especially in light of the continued technical advancements that make it ever more versatile and easier to use. ^1^ The fields of orthotics and prosthetics (O&P) are increasingly embracing the benefits of 3D printing,^2^ and there are many exciting developments^3^ which may help make established approaches more economical and – importantly – allow a fresh look at previously unsolved clinical issues, including problems such as lacking access to care, limited customizability, and restricted functionality. Of these issues, access to care is a major one, especially for patients in low-income countries with insufficient medical systems,^4^ but also in North America, where shortages of qualified labor can be predicted based on the age profile of the practitioner population and the trajectories of disease rates for conditions such as diabetes in the general population.^5, 6^

There is a recognized bottleneck in growing the O&P profession, namely, the rate at which new practitioners are trained in the various educational institutions. There were, at last count, 14 accredited O&P master programs offered across the United States,^7^ in addition to two in Canada, altogether representing the capacity of graduating perhaps 400 new professionals each year. This is perilously close to the replacement rate, considering that of some 14,000 active practitioners, assuming an average career duration of 35 years, an average of 400 are reaching retirement age each year as well. Against this background, the field can barely afford to have any O&P student either fail to graduate or, upon entering the workforce, switch into a different career or become otherwise lost to the profession.

Recruiting students who are both passionate about the profession and well prepared to be successful in it is a key to mitigating those issues. Ironically, the aforementioned buzz around 3D printing may be one vehicle to help with student engagement and recruitment. O&P is still a somewhat obscure field and is not at front of mind as a viable career choice for many high school or college students pondering their professional future. This is supported by anecdotal evidence: a recurring theme in the essay portions of most applications to O&P graduate programs is how the applicant has only by fortuitous happenstance become aware of the existence of the profession. This generally led to rapidly realizing its various exciting characteristics, including the ability to directly help patients, working with a broad range of materials and techniques, and the potential to have an impact and contribute to the advancement of the small field. While difficult to quantify, it is safe to assume many students do not apply for O&P graduate schools simply because they are unaware of the field and its profound impact on the lives of patients. Of course, having additional applicants would allow for more selective admission processes and/or expanded class sizes, which increases both the quality and quality of graduates to meet the needs of the field.

Again, based on the personal experience of one author (GF), media reporting on applications of 3D printing (a much more commonly known subject) for creating prostheses has often delivered a “lightbulb moment” for introducing young people to O&P (if the journalist had gotten around to reporting that there is indeed a specialized profession and not just hobbyists providing these devices!). There may therefore be ways of actively bridging connections with related technologies such as 3D printing to increase the applicant pool. In the university context, this includes offering introductory classes aimed at graduate and undergraduate from compatible backgrounds. For instance, students in medical engineering or bioengineering programs are often quite receptive to the idea of entering O&P when they are exposed to it in a practical way, often having had very compatible motivations for starting their engineering studies, including being drawn to problem-solving and helping people, and interest in novel technologies. With a strong background in physics, biology, materials science, problem-solving, design and prototyping, bioengineering students are also quite well prepared to succeed in the O&P curriculum. The first step could be an elective class offering that helps them fulfil some of the requirements for their undergrad degree while effectively introducing them to O&P. The authors have recently developed and pilot-tested such a class with promising success and believe that this approach could be adopted in other institutions as well.

## METHODOLOGY

The three-credit course was divided in two distinct halves: 1) a theoretical part to introduce the students to the basics of prosthetic management of limb loss, including the typical patient profile, prosthetic componentry, and workflows in the clinic in the first seven weeks of the semester, and 2) a practical part that was dedicated to designing and fabricating an actual prototype device in small group work, capped by final presentations/demonstrations in the remaining seven weeks. Beyond the lecture hall, class meetings were scheduled in the various fabrication laboratories within the institution, including the 3D-printing lab and the O&P teaching lab. Guest speakers were invited for several sessions to round out the covered content and provide interesting perspective. A written exam was administered at the midterm point and factored in the grade composition along with short quizzes and the final project reports and presentations.

We decided early in course design that, while computer-aided design (CAD) and 3D printing would be core enabling technologies presented in the course, we wanted the prime focus of our students' learning experience to be people who have unmet needs in prosthetic design. Putting a person at the center of the learning and design process is foundational to user-centered design or human-centered design,^8,9^ and stands in contrast to approaches that center a particular technology or technical method in the learning process. We adopted a design thinking approach which emphasizes empathy, deep investigation of problems before pursuing solutions, broad ideation before converging on trial solutions, early and frequent prototyping of trial solutions, and an iterative, empirical, evidence-based approach to making design decisions (rather than making choices based on untested and aspirational assumptions).

We particularly emphasized the importance of problem discovery as an essential and foundational tool to achieving success in creating a solution that will ultimately be effective for a user or client in real-world circumstances. Problem discovery involves first determining what is the “right” problem or problems to solve among the myriad possible problem formulations that may arise in a real-world design context. This process exercises and leverages basic human skills like talking to another person and learning to actively listen in ways that develop empathy between designer and client, and ideally actively engages the client as a co-designer in the solution-finding process, rather than as a “subject”.

**Figure 1:**
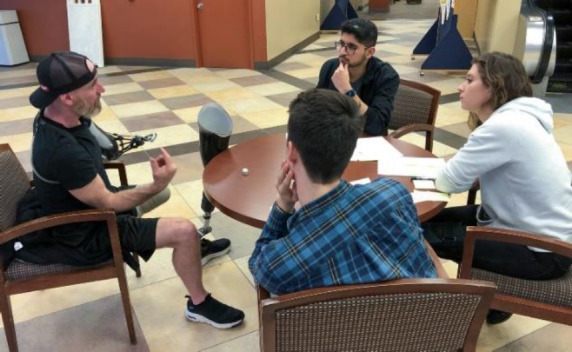
A student team interviews a user of prostheses at the start of a process of understanding unmet needs and discovering problems to design solutions for using a human-centered methodology.

To implement these concepts and methods from design thinking in our course in a practical and impactful manner, we arranged for three users of prosthetics (clients of author GF) to participate in the 3rd weekly class session and then continue to work with our students during the 14-week semester. To increase the impact and student engagement of this introductory experience, we employed a “dramatic reveal” of these clients to the students during a class session. This enabled the students to practice skills in listening, interviewing and problem discovery within minutes of the topics being introduced in the classroom. In the first hour of this class session the students received an introduction to design thinking, with illustrative examples provided for assistive technology applications. This was followed by an overview of problem discovery and an introduction to interviewing techniques. The students engaged in a preliminary exercise in which they interviewed each other. We then informed the students they would soon be able to apply the interviewing methods they just learned to better understand how to improve the prosthesis experience for several actual users of prosthetics – at which point we immediately welcomed our three clients into the classroom. After they briefly shared their backgrounds with the students, we formed multiple teams each consisting of one client and several students. These teams then spent about an hour in small-group discussion, providing the start of our students' needs-finding process (**Figure 1**).

We provided three prompts to our student teams before they began their interviews:

- Before we interviewed our client/design partner, we thought:
- After our interview, we now know:
- One thing we were surprised to learn was:

Each team then shared their answers to the above prompts during a wrap-up class discussion. We were highly impressed by the insights shared by the students, often indicating ways their assumptions going into the interviews had been significantly changed by speaking with an actual user of a prosthesis. Feedback from the students was also overwhelmingly positive, with many indicating this experience marked a watershed moment in their motivation toward the course content and their enthusiasm for engaging in new prosthetic designs to address specific client needs.

## RESULTS

**Figure 2:**
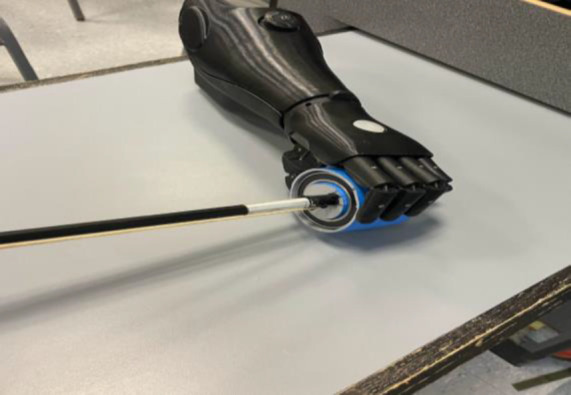
Cello Bow holding adapter.

**Figure 3:**
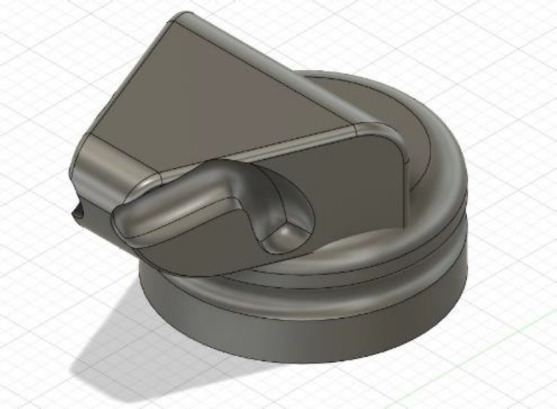
Stick-shift to prosthetic hook adapter.

While the limb loss levels of the invited patient models – in this case including individuals with trans-femoral, trans-radial, and trans-humeral amputations as well as one with congenital hand defect – somewhat predetermined the nature of the student-designed devices, it was deliberately left an open-ended question what clinical need exactly each group would identify and try to solve. Eventually, the four group projects in this iteration of the course were (A) an adapter to improve grip on a cello bow for the user of a myoelectric hand (**Figure 2**), (B) an adapter to facilitate safe operation of a manual-shift sportscar for the user of a body-powered prosthetic arm (**Figure 3**), (C) a prosthesis-compatible elastic sleeve with vibration motors for pain relief for a trans-femoral prosthesis user, and (D) a self-leveling prosthetic wrist joint intended to improve safe carrying of open containers. Projects were evaluated using a grading matrix with the criteria Design Selection, Prototype Development, Documentation, and Justification (**Table 1**). All students easily received passing grades.

**Table 1:** Grading rubric for project report (adapted, with permission, from knowlesteachers.org^10^)

Criteria	4 points	3 points	2 points	1 point
	Advanced - Exceeds expectations	Competent - Meets expectations	Progressing - Does not fully meet expectations	Beginning - Does not meet expectations
Design selection: can compare a range of design concepts, and select a preliminary design that best meets the identified constraints and criteria	Deliberately and effectively uses initial testing, data and/or research to objectively support preliminary design selection. Defends preliminary design choice against other concepts in light of criteria and constraints (trade-offs) using an appropriate objective tool (e.g., decision matrix).	Deliberately uses initial testing, data and/or research to subjectively support preliminary design selection. Defends preliminary design choice against other concepts in light of criteria and constraints (trade-offs).	Uses data unsystematically for preliminary design selection. Selects preliminary design based on criteria that are poorly aligned with criteria or constraints.	No data collected to support preliminary design selection. Evidence for preliminary design choice not logical or unfounded (choices made without rationale, or based on untested assumptions or “favorite” concepts)
Prototype development: demonstrates form and functionality of the design by creating a working prototype.	Prototype meets all constraints. Prototype functionality exceeds expectations of detailed final design. Prototype effectively communicates the form of the detailed final design with professional level quality.	Prototype meets all constraints. Prototype functionality matches detailed final design. Prototype effectively communicates the form of the detailed final design, and exhibits appropriate quality/craftsmanship	Prototype meets most but not all constraints. Prototype functionality partly matches expectations of detailed final design. Prototype roughly communicates the form of the detailed final design.	Prototype meets few constraints. Prototype is insufficient to demonstrate basic functionality of detailed final design. Prototype does not communicate the basic form of the detailed final design.
Documentation: create a documentation package that clearly explains the detailed final design and corresponding testing/validation results.	Design documentation is appropriately detailed and structured for the intended purpose and audience; extraneous information has been removed. Documentation includes tolerances for all necessary specifications. Documentation is polished and professional.	Design documentation is appropriately detailed and structured for the intended purpose and audience. Documentation is sufficiently organized and includes all necessary specifications for assembly and/or operation. Documentation is well-organized, professional, and free of mechanical errors.	Design documentation is detailed but may not be optimized for the designated purpose. Documentation is organized and includes most of the key parameters for assembly and/or operation and contains few mechanical errors.	Design documentation is not appropriate for the designated audience. Documentation lacks crucial information. Documentation requires significant editing and/or formatting.
Justification: can explain the benefits and weaknesses of the design, including opportunities, tradeoffs and ideas for further improvement	Communicates the design's strengths and limitations relative to competitor benchmarks and other design options. Evaluates design as well as opportunities and tradeoffs in light of criteria and constraints and defends the validity of metrics used. Recommends design improvements which are supported by objective evidence or data.	Communicates the design's strengths and limitations relative to other design options. Evaluates design as well as opportunities and tradeoffs in light of criteria and constraints. Recommends design improvements which are supported by subjective evidence.	Communicates the design's strengths relative to other design options. Evaluates design based on criteria and constraints. Recommends design improvements; no evidence is cited to support these recommendations.	Does not consider other design options. Does not cite the criteria and constraints in evaluation of design. No suggestions for improvement are offered.

Feedback on the course, both from the students and the patient models, was positive throughout. All enjoyed the collaborative and creative atmosphere. The engineering students were especially appreciative of the opportunity to work with the actual user of their designs and clearly were motivated to put forth their best effort. The ability to observe the direct results of their work provided a sense of achievement and some memorable moments, for instance, when on one occasion the entire lab quieted down to listen to our cellist patient play a beautiful tune in testing his new bow adapter.

Structured student feedback on the class experience, as is routinely solicited by the institution was encouraging as well, with an overall rating of 4.89 on a 0–5 scale. Positive student comments included statements like: “Overall, this was a great course that taught me essential hands–on skills that I have yet to learn in my engineering coursework. Working with patient models was also an experience that I am very grateful to have after taking this course” and “Bringing in the patient models and introducing them to us is super helpful.” Where students noted room for improvement, they wished for more time to be allocated to the design and prototyping work and that a nominal budget for material purchases be provided.

## CALL TO ACTION

Having an O&P education program housed within a university offers great opportunity for the recruitment of talented young engineers into the next generation of practitioners. We believe it is time well spent for O&P faculty to offer respective coursework, especially when they can team up with colleagues from other departments to do so. The popularity of 3D printing among students can be leveraged by designing a class to highlight this central topic as an enabling technology while centering the overall design process in a human-centered approach that maintains a top-level emphasis on the people whose unmet needs will be explored and serve as the focus for solution discovery. Students may initially come for the 3D printing but stay for the experience of working with patients how only O&P can provide it. We encourage all colleagues to propose and provide such coursework where possible.

## DECLARATION OF CONFLICTING INTERESTS

None.

## AUTHORS CONTRIBUTION

Both authors contributed equally to the research and the writing of this manuscript.

## SOURCES OF SUPPORT

“Classroom to Community: Designing and Inventing for Real-World Impact,” Pitt Seed Grant, University of Pittsburgh.

## AUTHORS SCIENTIFIC BIOGRAPHY

**Figure:**
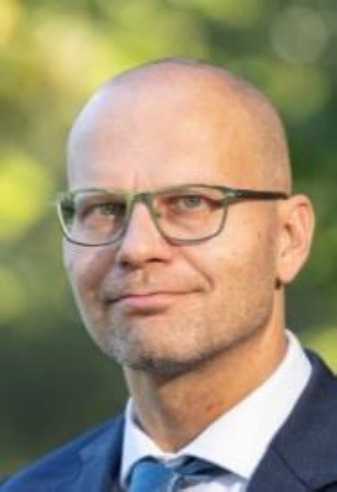


**Goeran Fiedler**, PhD, is an associate professor for the Master of Science in Prosthetics and Orthotics program at the University of Pittsburgh. He holds graduate degrees in Clinical Engineering/Biomechanics from the University of Applied Sciences Giessen (Germany) and in Health Sciences from the University of Wisconsin-Milwaukee. He completed his post-doctoral training in the Department of Rehabilitation Medicine at the University of Washington in Seattle. He is also a credentialed Prosthetist & Orthotist with degrees from the German Chambers of Craft in Thuringia and in Lower Bavaria, as well as 12 years of clinical work experience. His research and teaching have the overall goal to find ways of raising the quality and quantity of prosthetic & orthotic device utilization, in order to realize outcome gains that are inexpensive to achieve and applicable to a large patient population.

**Figure:**
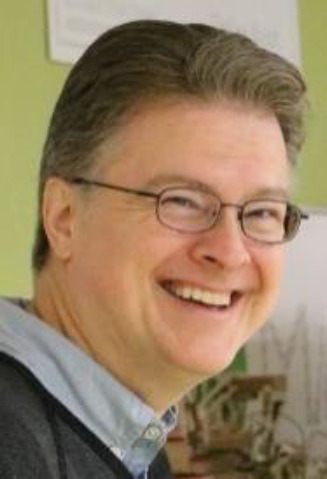


**Joseph Samosky**, PhD, is Associate Professor of Bioengineering in the Swanson School of Engineering at the University of Pittsburgh. He received his PhD in Medical Engineering from MIT in the Harvard-MIT Program in Health Sciences and Technology. Over the past decade he has designed, developed and deployed new courses to foster user-centered design, innovation, creativity and project-based learning. Dr. Samosky has mentored over 1000 students in more than 200 design and innovation projects and his students have received numerous awards in design and innovation competitions. He has 8 issued patents to date. He also developed and directs the Swanson School of Engineering's G34 Design and Innovation Space, a student-centered home base for creative community, ideation, prototyping and invention. He received the School of Engineering's Outstanding Educator award in 2018 and was named Higher Educator of the Year in 2023 by the Engineers Society of Western Pennsylvania.
